# Camptothecin Delivery *via* Tumor-Derived Exosome for Radiosensitization by Cell Cycle Regulation on Patient-Derived Xenograft Mice

**DOI:** 10.3389/fbioe.2022.876641

**Published:** 2022-04-12

**Authors:** Yiling Yang, Shiqi Ren, Wenpeng Huang, Jiahan Dong, Jiancheng Guo, Jie Zhao, Yonggao Zhang

**Affiliations:** ^1^ Department of Ultrasound, The First Affiliated Hospital of Zhengzhou University, Zhengzhou, China; ^2^ BGI College and Henan Institute of Medical and Pharmaceutical Sciences, Zhengzhou University, Zhengzhou, China; ^3^ Department of Radiology, The First Affiliated Hospital of Zhengzhou University, Zhengzhou, China; ^4^ Internet Medical and System Applications of National Engineering Laboratory, Zhengzhou, China

**Keywords:** cell cycle, exosome, radiotherapy, patient-derived xenograft model, camptothecin

## Abstract

**Purpose:** While radiotherapy remains the leading clinical treatment for many tumors, its efficacy can be significantly hampered by the insensitivity of cells in the S phase of the cell cycle to such irradiation.

**Methods:** Here, we designed a highly targeted drug delivery platform in which exosomes were loaded with the FDA-approved anti-tumor drug camptothecin (CPT) which is capable of regulating cell cycle. The utilized exosomes were isolated from patient tumors, enabling the personalized treatment of individuals to ensure better therapeutic outcomes.

**Results:** This exosome-mediated delivery strategy was exhibited robust targeted to patient-derived tumor cells *in vitro* and in established patient-derived xenograft models. By delivering CPT to tumor cells, this nanoplatform was able to decrease cell cycle arrest in the S phase, increasing the frequency of cells in the G1 and G2/M phases such that they were more radiosensitive.

**Conclusion:** This therapeutic approach was able to substantially enhance the sensitivity of patient-derived tumors to ionizing radiation, thereby improving the overall efficacy of radiotherapy without the need for a higher radiation dose.

## Introduction

Cervical cancer is among the leading causes of cancer and cancer-associated mortality among women (Sung et al., 2021). In individuals with stage Ib and IIa cervical cancer, radiotherapy and radical surgery can both effectively improve patient outcomes, yielding satisfactory 5-year survival rates (Landoni et al., 1997). However, these approaches are less efficacious in more advanced cervical cancer patients in whom tumors are larger (Rose et al., 1999). To achieve the desired outcome, the utilized ionizing radiation dosage may exceed maximum normal tissue tolerance thresholds, resulting in severe adverse events (Sasieni and Sawyer 2021). To overcome this risk, other therapeutic methods including chemotherapy, afterloading therapy, and radioactive particle implantation have been explored (Flay and Matthews 1995).

Nanomedicine-based therapeutic radiosensitization strategies represent a novel emerging approach to enhancing the efficacy of radiotherapy while minimizing normal tissue damage (Zhang et al., 2019; G.; Song et al., 2017). Nano-radiosensitizers have been developed that are capable of increasing ionizing energy deposition in target tumor tissues by enhancing the photoelectric effect. These radiosensitizers are generally composed of high-Z elements including silver (Khochaiche et al., 2021), gold (Yi et al., 2016; Laprise-Pelletier et al., 2018; Xu et al., 2019), tantalum (Song et al., 2016; Lyu et al., 2021), and hafnium (Chen et al., 2019). While promising, however, these nano-radiosensitizers exhibit intrinsic cytotoxicity, and their metabolic processing remains uncertain (Yoon et al., 2018). Other approaches have sought to leverage the tumor microenvironment (TME) to increase radiosensitivity (Jarosz-Biej et al., 2019; Chen et al., 2021). For example, catalytic agents capable of converting H_2_O_2_ to O_2_ within cells have been used to alleviate intratumoral hypoxia as a means of improving radiotherapeutic outcomes (Zhu et al., 2016; Lin et al., 2018; Lyu et al., 2020a). Researchers have also sought to combine radiotherapy with chemotherapy, photothermal therapy, and sonodynamic therapy in an effort to achieve synergistic benefits superior to those of either treatment in isolation (Lyu et al., 2020b; Suo et al., 2020; Yang et al., 2020). Chemotherapeutic drugs can be broadly classified into cell cycle-specific and -nonspecific agents, with the former of these inducing apoptotic cell death at a particular point in the cell cycle and the latter resulting in indiscriminate target cell death (Zhu et al., 2020; Luo et al., 2020). Chemotherapy is a systemic treatment, in contrast to radiotherapy, which is targeted. Severe off-target side effects of chemotherapy can include nausea, malaise, and alopecia (Rose et al., 1999; Bosset et al., 2006; Abraham et al., 2013; Gao et al., 2020; Khochaiche et al., 2021; Zhu et al., 2022). Careful consideration of the most appropriate drug delivery strategy must thus be considered to maximize benefit and minimize discomfort in treated cancer patients (Peeters et al., 2005).

**SCHEME 1:**
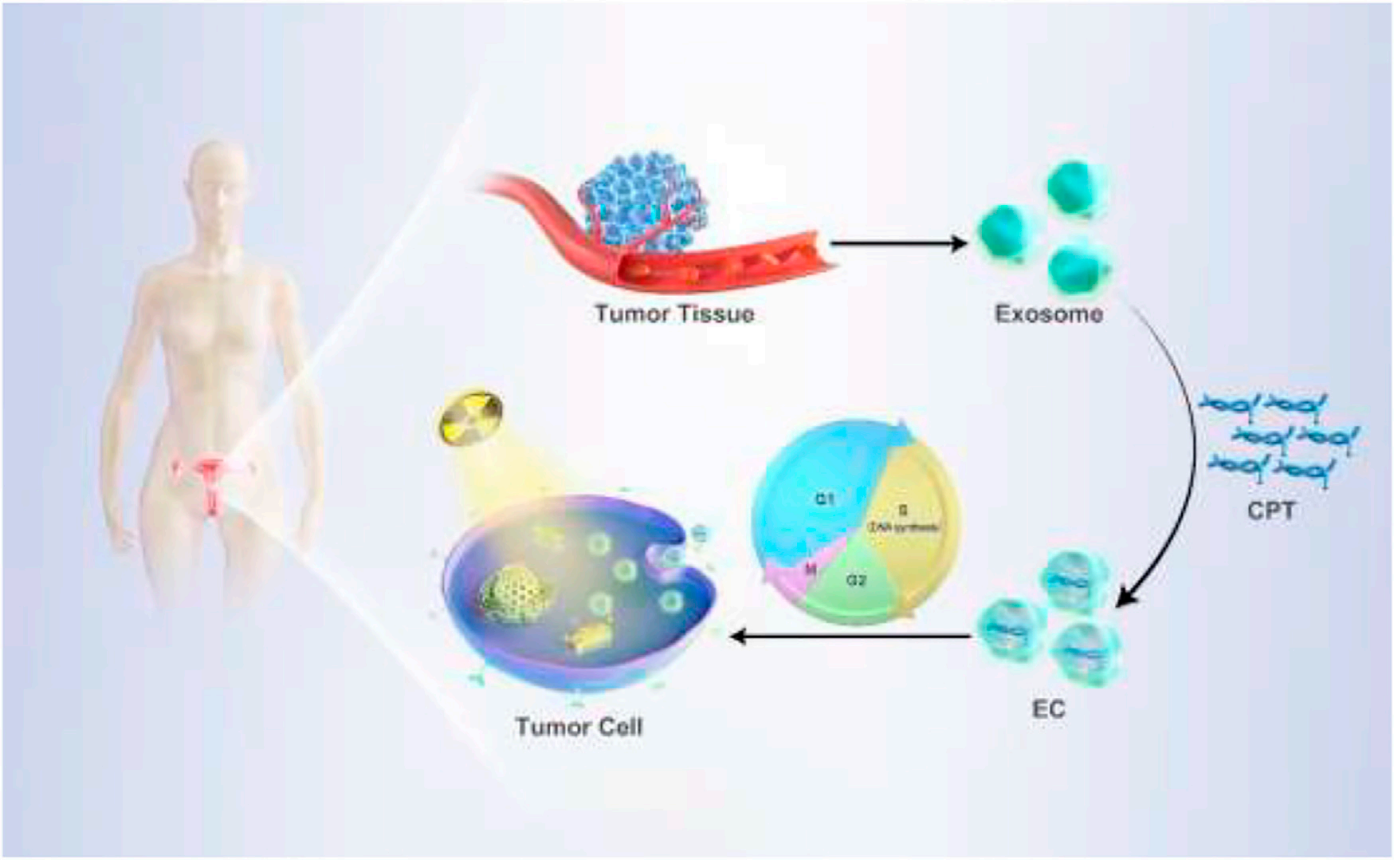
Personalized treatment procedure on PDX model using EC.

In recent years, personalized therapeutic medicine-based approaches have emerged for the treatment of cancers (Wang et al., 2021). In most *in vivo* preclinical assays, researchers rely on cell-line-derived xenograft (CDX) murine models derived from purified cell lines. While informative to some extent, these models differ substantially from natural tumors. Patient-derived xenograft (PDX) models, in contrast, utilize tumor tissues derived directly from patients and thus more closely recapitulate the heterogeneity, structure, and metastatic potential of true human tumors (Sun et al., 2021). In the present study, we utilized tumor-specific patient-derived exosomes as an approach to delivering camptothecin (CPT) to tumors in PDX model mice (Bhimani et al., 2020). As shown in Scheme 1 CPT is a chemotherapeutic drug capable of reducing the frequency of cells in the S phase of the cell cycle during which DNA replicates and cells are largely resistant to ionizing radiation (Park et al., 1997). Owing to their robust tumor-targeting capabilities, patient-derived exosomes from a cervical cancer patient were utilized as a vehicle to mediate CPT delivery to the target tumor, thereby enhancing radiosensitization while minimizing normal adjacent tissue damage (Duo et al., 2021). In addition, exosomes derived from HeLa cells were used to deliver CPT, and the relative feasibility of both patient-derived exosome/CPT (EC) hybrid and HeLa cells exosome CPT (ECC) hybrid nanoplatforms were analyzed in a PDX model system using tumor tissues from the same cervical cancer patient. Remarkable, the developed EC hybrid nanoplatform strategy exhibited efficient tumor targeting together with robust radiosensitization, thus underscoring its promising biocompatibility and translational potential.

## Experimental Section

### Animals

Six-week-old female nude BALB/c mice were purchased from Vital River Company (China). The protocols of the Institutional Animal Care and Use Committee were used to guide all animal studies discussed herein.

## Patient Tumor Samples

Tumor tissue samples were collected from a 55-year old female patient with advanced stage IIIc cervical cancer within 1 h of surgery as per the protocols of the Institutional Review and Ethics Boards of Zhengzhou University. The patient provided informed consent for the present study.

### Patient-Derived Cancer Cell Isolation

Initially, tumor tissues were minced into small ∼1 mm^3^ cubes, after which they were digested for 1 h using 0.1 mg/ml collagenase IV and filtered through 70 um strainers (Becton Dickinson, United States) to yield patient-derived cancer cells.

### EC and ECC Synthesis

Patient-derived exosomes were prepared as per a previously described ultraviolet (UV) stimulation method (Tang et al., 2012; Wang et al., 2019). Briefly, patient-derived cancer cells were plated and exposed to UV irradiation (300 J/m^2^) for 1 h. Then, after 12 h, cells were centrifuged at 140,000 g for 2 min to remove any cellular debris, and supernatants were again centrifuged for 1 h at 140,000 g to pellet patient-derived exosomes. Pellets were then washed thrice and resuspended in culture media for subsequent experimental use. EC preparation was conducted by mixing patient-derived exosomes (0.5 mg) and CPT (10 μg in 10 μL DMSO) in 250 μL of PBS in a 0.4 cm cuvette (Bio-Rad), with electroporation (250 V and 350 μF) then being conducted using a Bio-Rad Gene Pulser Xcell Electroporation System. Membrane integrity was then allowed to recovery through a 30 min incubation at 37°C, after which samples were spun for 1 h at 140,000 g to remove unincorporated CPT. EC samples were then stored at 4 °C. ECC preparation was conducted *via* an identical approach, instead using exosomes derived from HeLa cells. Drug loading efficiency (DLE) was calculated as follows: DLE = (weight of input drug—weight of remaining drug)/weight of input drug.

### Animal Model

A PDX model was established using previously reported methods. Briefly, harvested patient tumor tissues were minced into ∼5 mm^3^ pieces within 1 h of surgery, after which they were subcutaneously implanted in nude BALB/c mice with a trochar (termed P0). At 2–3 months post-implantation, tumors began to visibly grow. When tumors were 800–1,000 mm^3^ in size, mice were euthanized and tumors were collected, respected, and implanted as above to yield a subsequent generation (P1). This process was repeated two more times, with P3 mice being used for all *in vivo* analyses.

### Antitumor Efficacy

When tumors in P3 mice were 200 mm^3^ in size, mice were randomized into six groups subjected to the following treatments: 1) PBS; 2) RT (6 Gy); 3) CPT (5 mg/kg, 50 μL); 4) EC (equivalent CPT dose: 5 mg/kg, 50 μL); 5) ECC (equivalent CPT dose: 5 mg/kg, 50 μL) + RT (6 Gy); 6) EC (equivalent CPT dose: 5 mg/kg, 50 μL) + RT (6 Gy). Tumor volumes and weight were then monitored every third day, with mice being sacrificed on day 19. Tumors were then collected for immunofluorescent and hematoxylin and eosin (H&E) staining.

## Results and Discussion

**FIGURE 1:**
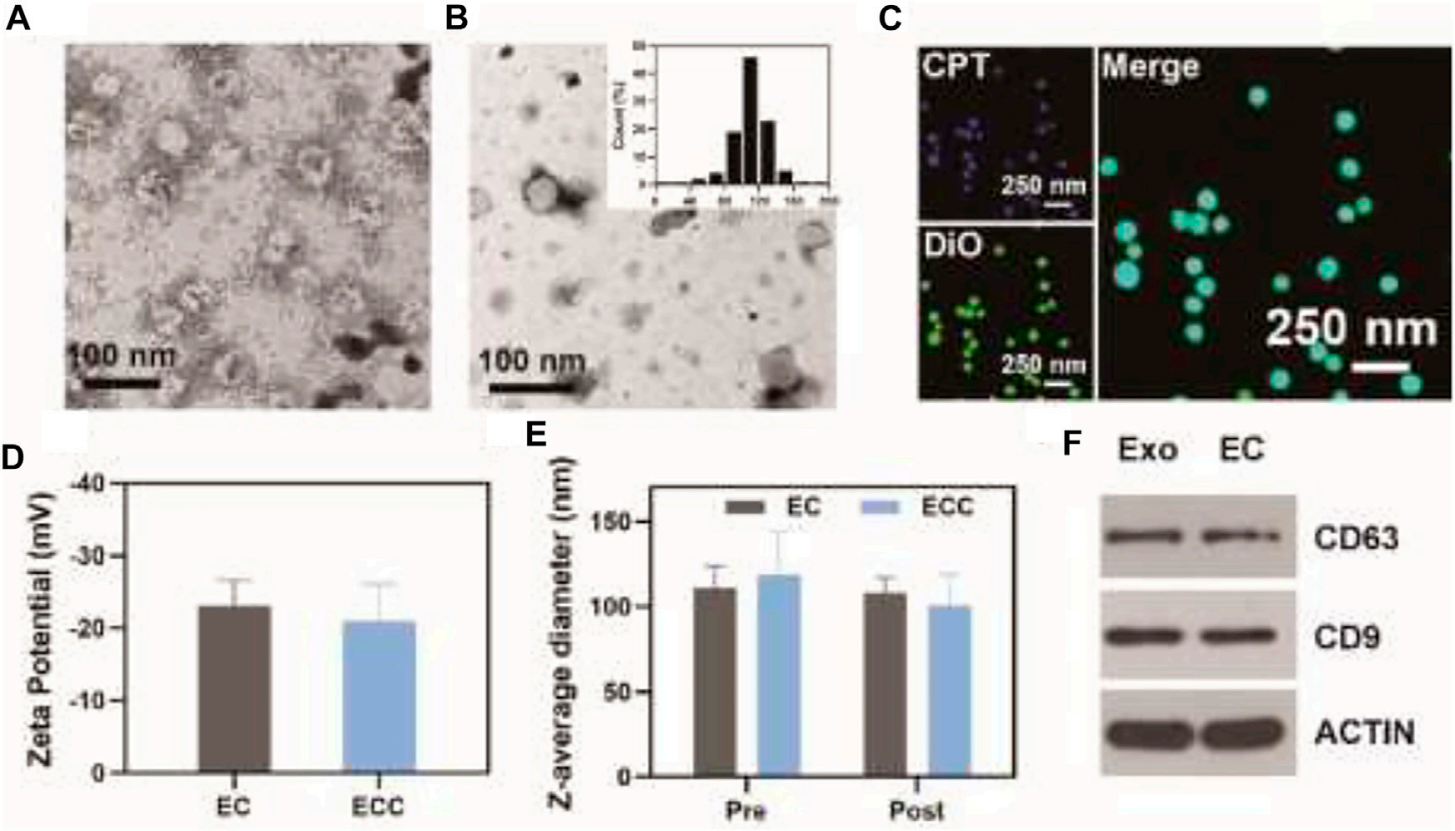
Exosome characterization. **(A)** ECC and **(B)** EC exosomes were imaged *via* transmission electron microscopy, with the inset graph showing EC diameter distributions. **(C)** Confocal microscopic analysis of the colocalization of CPT (blue) and DiO (green) within the EC. **(D)** EC and ECC Zeta potential values; **(E)** EC and ECC Z-average diameter values before and after irradiation (6 Gy); **(F)** Western blotting analysis of patient-derived exosomes and EC.

Patient- and HeLa cell-derived exosomes were initially prepared *via* UV stimulation and were then loaded with CPT *via* an electroporation approach. EC and ECC particles exhibited an expected round morphology with a visible lipid layer and a diameter of ∼115 nm (Figures 1A,B). Confocal microscopy analyses were conducted to confirm successful CPT loading into these exosomes using 3, 3-dioctadecyloxacarbocyanine perchlorate (DiO) as a fluorescent membrane probe together, with CPT also being visualized as it releases bright blue fluorescence at an excitation wavelength of 365 nm, with both of these fluorescent signals being visible in prepared revealing both blue CPT and bright green DiO fluorescence confirming the coupling reaction (Figure 1C). CLSM imaging further confirmed the loading of CPT into HeLa cell-derived exosomes (Supplementary Figure S1). Moreover, the DLE of EC and ECC were 23.5 and 21.5% respectively (Supplementary Figure S2). DLS was used to calculate zeta potential values for EC and ECC preparations in PBS (Figure 1D). Stability in response to irradiation is a key clinical concern when exploring the potential biomedical application of nano-radiosensitizing agents. To assess EC and ECC stability following radiotherapy treatment, Z-average diameter values were measured for these particles before and after treatment (Figure 1E), with the observed absence of any apparent treatment-related change being indicative of satisfactory stability. Western blotting was additionally conducted for patient-derived exosomes and ECC samples to confirm their composition. Both of the patient-derived exosomes and ECC samples exhibited high levels of expression of the exosomal marker proteins CD9 and CD63, indicating the existence of exosomes in ECC samples (Figure 1F). As such, these results confirmed the successful production of patient- and tumor cell-derived exosomes loaded with CPT.

**FIGURE 2:**
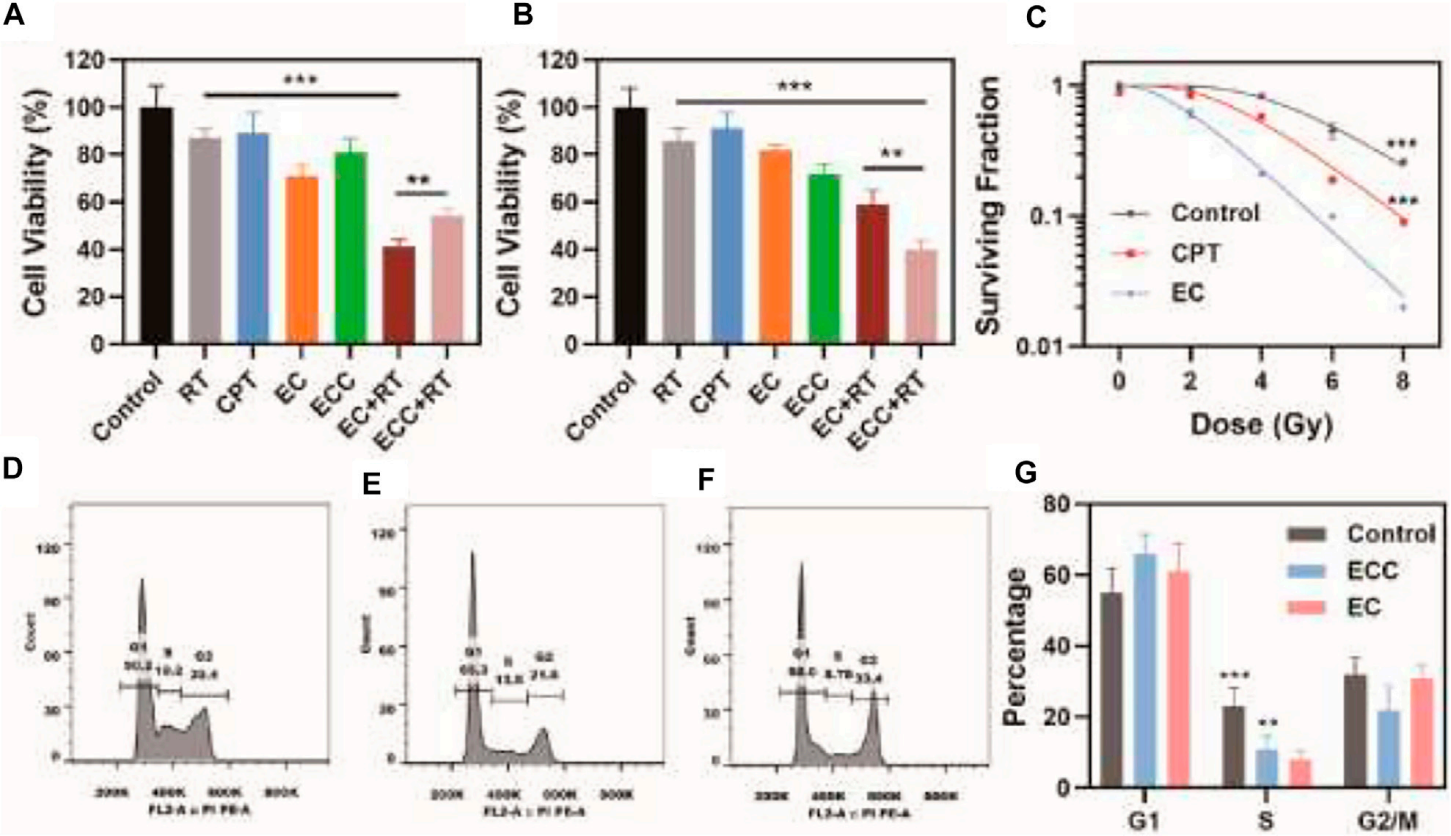
Assessment of *in vitro* therapeutic efficacy. **(A)** Patient-derived cell and **(B)** HeLa cell viability following the indicated treatments. **(C)** Colony formation assay. **(D–G)** Flow cytometry was used to assess cell cycle progression in patient-derived cells treated with **(D)** PBS, **(E)** ECC, and **(F)** EC, with a **(G)** corresponding quantitative cell cycle analysis. *p < 0.05, **p < 0.01, ***p < 0.005; Student’s t-test.

EC and ECC biocompatibility were next assessed using the Ect1/E6E7, End1/E6E7, Vk2/E6E7 and HUCEC cervical epithelial cell line, revealing that both of these preparations were associated with negligible cytotoxicity even at high concentrations (Supplementary Figure S3). To evaluate EC- and ECC-associated radiotherapy-induced apoptosis, a CCK-8 assay was used to evaluate cell death for both HeLa and patient-derived cells treated with these preparations. While cells treated with RT or CPT alone exhibited >85% viability (Figure 2A), consistent with poor efficacy, and EC or ECC treatment alone had limited inhibitor effect, combined ECC + RT and EC + RT treatment were associated with reductions in cell viability to 54.2 and 41.2%, respectively, consistent with robust combination therapeutic efficacy. This efficacy may be attributable to the specific targeting capabilities of patient-derived exosomes. Similar results were also obtained for HeLa cells (Figure 2B), with ECC + RT treatment yielding more robust tumor inhibition than EC + RT treatment in this assay system, likely owing to the share homology between HeLa cells and exosomes derived therefrom. Even so, these results suggest that EC and ECC preparations both exhibit some degree of cross-reactive homology that is beneficial to cervical tumor targeting.

Colony formation assays were next conducted to assess the ability of ECC and EC preparations to sensitize patient-derived cells to radiotherapy, revealing clear differences in the ability of EC- and ECC-treated cells following irradiation (Figure 2C), consistent with more robust radiosensitization activity. Specifically, EC and ECC exhibited sensitivity enhancement ratio values of 1.13 and 1.35 respectively.

CPT is a cell cycle-regulating antitumor drug. As such, flow cytometry was next used to assess cell cycle progression in patient-derived cells exposed to PBS, ECC, and EC (Figures 2D−F). ECC treatment was associated with a decrease in the frequency of cells in S-phase as compared to the control group, and this difference was even more significant for EC-treated cells. Indeed, quantification of these results revealed that the percentages of cells in the S phase in the EC, ECC, and control groups were 8.1, 11.0, and 22.9%, respectively (Figure 2G). As such, these results confirmed the ability of EC treatment to enhance radiosensitivity owing to the ability of patient-derived exosomes to efficiently deliver CPT to target tumor cells in which it was able to modulate cell cycle progression. Moreover, as shown in Supplementary Figure S4, Cdk2 expression exhibited an pbvious decrease after treatment with EC while a slight decrease of Cdk2 in group treated with ECC, which indicated that EC is capable of regulating cell cycle since the content of Cdk2 raises in the S phase however decreases in G2/M phase. Both of ECC and EC group exhibite great improvement in p-H3 expression, which reflects mitotic abnormalities. It could also be found that p21 and cyclin B1 expression significantly increased in both group treated with ECC and EC, the latter of which demonstrated a more obvious enhancement, suggesting ECC and EC inhibite the activity of Cdk in G2/M.

**FIGURE 3:**
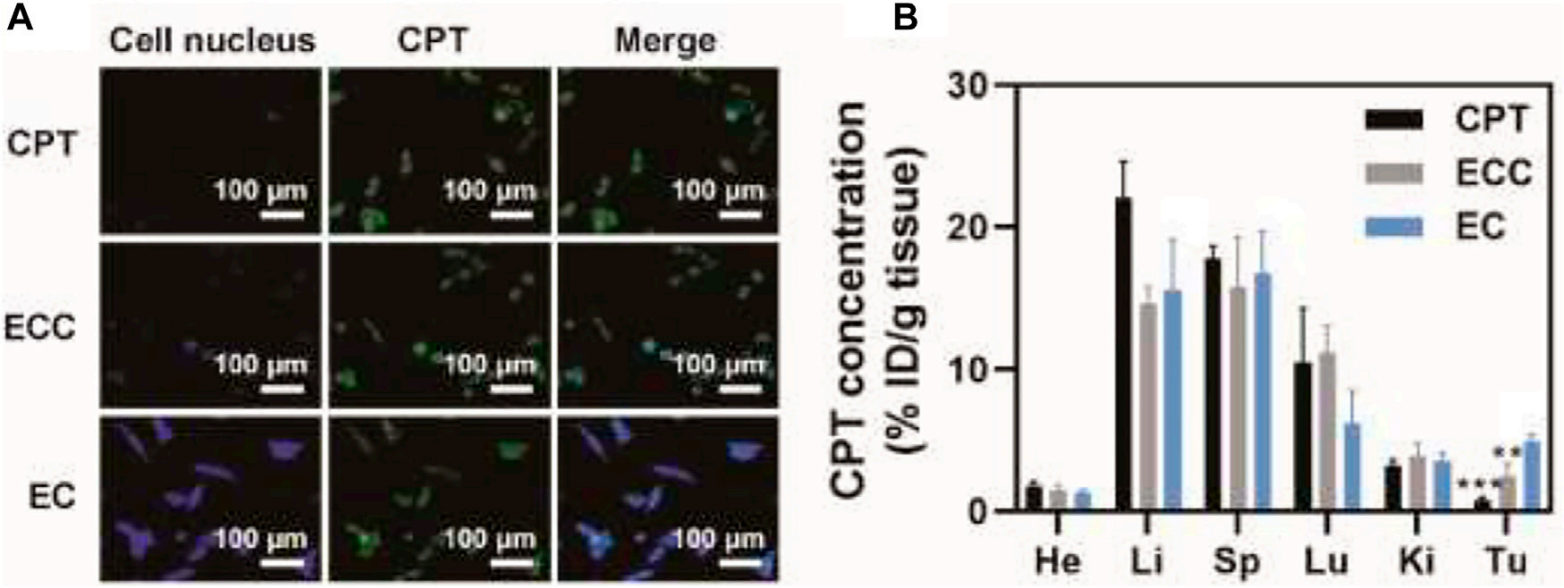
Assessment of tumor-targeting capabilities. **(A)** CLSM images of patient-derived cells following CPT, ECC, and EC treatment, with CPT and mitochondria respectively shown in blue and green. **(B)** CPT biodistribution in mice intravenously administered with CPT, EC, and ECC. *p < 0.05, **p < 0.01, ***p < 0.005; Student’s t-test.

Given the above results and to further confirm the ability of ECC to facilitate specific tumor targeting, patient-derived tumor cells were treated for 1 h with CPT, ECC, or EC preparations. CLSM was then performed, with mitochondria being stained using the MitoTracker green probe, to assess CPT uptake. EC-treated cells exhibited greater fluorescence intensity as compared to cells in the ECC group, while both of these treatments were associated with superior CPT internalization as compared to direct CPT treatment (Figure 3A), consistent with the ability of exosomal preparations to mediate drug internalization and mitochondrial accumulation. Then, a PDX mouse model was used to assess the *in vivo* targeting characteristics of EC preparations, with CPT biodistribution characteristics in major organs being quantified *via* high-performance liquid chromatography (HPLC). At 6 h post-injection, drug levels in the tumor reached ∼4.9% ID/g in the EC group (Figure 3B), with these levels being 6.1- and 1.9-fold higher than in the CPT (∼ 0.8% ID/g) and ECC (∼ 2.5% ID/g) groups, respectively. Higher tumor-to-normal tissue distribution ratios were observed for EC as compared to ECC and CPT, indicating the robust tumor selectivity of EC preparations. As such, these results confirmed that patient-derived exosomes can readily deliver CPT to target tumors *in vivo.*

**FIGURE 4:**
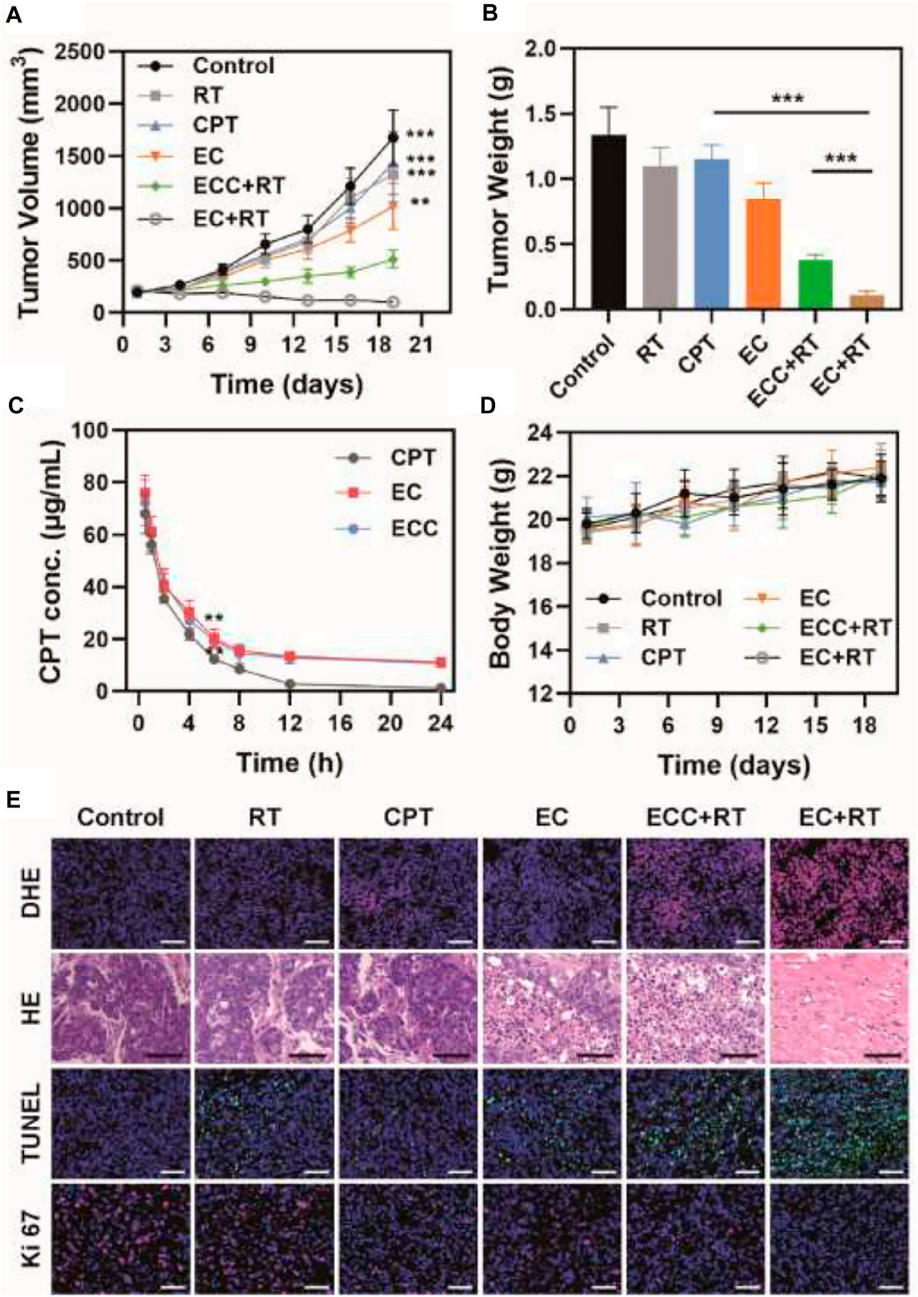
Analysis of antitumor efficacy. **(A)** Tumor volumes and **(B)** tumor weight values for mice in the indicated treatment groups. **(C)** Pharmacokinetics curves for mice injected with CPT, EC, and ECC. **(D)** Changes in murine body weight over the study period. **(E)** DHE, H&E, TUNEL, and Ki 67 staining were conducted after treatment in the indicated groups (Scale bar: 100 μm). *p < 0.05, **p < 0.01, ***p < 0.005; Student’s t-test.

Given the excellent *in vitro* cytotoxicity, cell cycle regulatory activity, and tumor cell accumulation observed for the EC nanoplatform in the above studies, we next assessed its ability to mediate *in vivo* therapeutic efficacy in nude mice bearing PDX tumors. To that end, mice were intravenously injected with different formulations (CPT, EC, ECC; CPT dose: 5 mg/kg) prior to irradiation (6 Gy). Tumor growth and tumor weight values in these treated animals were then monitored (Figures 4A,B). While tumors grew rapidly in PBS-treated mice, weak suppression of tumor growth was evident in mice treated with RT, CPT, or EC alone. In contrast, combination EC + RT treatment resulted in significant antitumor activity, with a 94.0 and 91.8% reduction in tumor volume and tumor weight, respectively, relative to the PBS group. This suppression was also superior to that observed in the ECC + RT group, and the overall tumor inhibition rate in the EC + RT group was 3.43-, 5.48-, and 1.32-fold higher than that observed in the RT, CPT, and EC groups, respectively. While ECC + RT treatment was also effective, its efficacy was less robust than that of EC + RT owing to reductions in tumor targeting efficiency and intratumoral exosome accumulation. Both EC and ECC exhibited prolonged systemic circulation as compared to CPT. At 6 h post-injection, CPT concentrations in the ECC and EC groups were 19.3 μg/ml and 20.3 μg/ml, respectively, owing to the ability of the exosomes to protect this drug from immune system-mediated clearance (Figure 4C). Notably, none of the tested treatments were associated with any significant systemic toxicity in these PDX tumor-bearing mice as determined by measuring murine body weight (Figure 4D).

Tumor tissue sections from mice in each treatment group were isolated to evaluate tumor tissue histopathological characteristics *via* immunofluorescent and H&E staining (Figure 4E). H&E staining revealed that PDX tumors exhibited a larger extracellular matrix component and more disordered cell distributions, thus more closely recapitulating findings observed in cervical cancer patients. Relative to the PBS, RT, and CPT groups, tumor cells in the other treatment groups (EC, ECC + RT, EC + RT) exhibited varying degrees of cellular shrinkage and nuclear pyknosis that was particularly pronounced in the EC + RT group. The reactive oxygen species (ROS) production, proliferation, and apoptosis of these cells were also assessed *via* DHE, Ki-67, and TUNEL, respectively. ROS levels were higher following EC + RT treatment relative to other treatments. Similarly, EC + RT treatment was associated with the greatest suppression of cellular proliferation (Ki-67 positive cells, red) and the greatest percentage of apoptosis cells (green fluorescence) relative to other tested treatments, consistent with the robust radiosensitization activity of EC.

**FIGURE 5:**
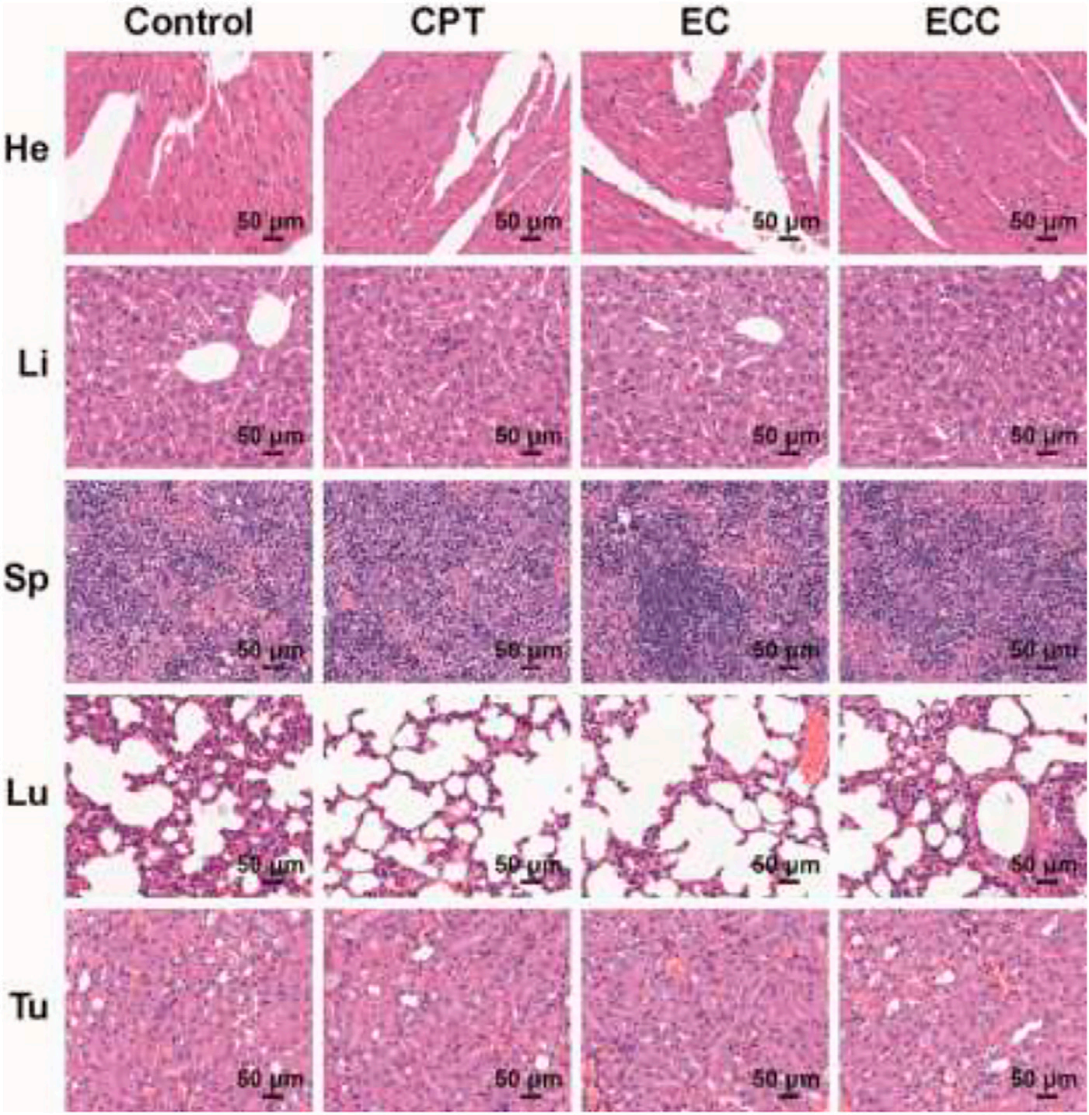
H&E staining of tumor samples from mice injected with PBS, CPT, ECC, and EC.

Systemic toxicity in treated mice was assessed on day 19 post-treatment by collecting the primary organs from animals intravenously injected with PBS, CPT, ECC, and EC for H&E staining. These analyses did not reveal any serious off-target toxicity in normal organs for mice in any of the tested treatment groups (Figure 5).

## Conclusion

In summary, we herein developed a patient-derived exosome CPT (EC) delivery platform as a personalized therapeutic modality for cervical cancer treatment. The prepared EC nanoplatform exhibited specific tumor targeting capabilities and consequent internalization owing to homology with the target cells. Hereby, the accumulation of antitumor drug CPT within these cells was enhanced. Therefore, it was able to modulate the cell cycle and thereby increase tumor cell sensitivity to radiotherapy. Through this approach, EC treatment was able to achieve marked antitumor efficacy both *in vitro* and *in vivo* when combined with radiotherapy without any concomitant systemic toxicity. As such, this therapeutic modality may offer promise as a personalized treatment for patients with a range of cancer types.

## Data Availability

The raw data supporting the conclusion of this article will be made available by the authors, without undue reservation.
