# Supplementary material for: Camptothecin Delivery via Tumor-Derived Exosome for Radiosensitization by Cell Cycle Regulation on Patient-Derived Xenograft Mice
Source: Front Bioeng Biotechnol. 2022 Apr 12;10:876641. doi: 10.3389/fbioe.2022.876641 (PMC9039187; doi:10.3389/fbioe.2022.876641)
Supplement: Supplementary file 1 [file DataSheet1.docx]

**Camptothecin delivery via tumor-derived exosome for radiosensitization by cell cycle regulation on patient-derived xenograft mice**

Yiling Yang^1^*, Shiqi Ren^2^,Wenpeng Huang^3^, Jiahan Dong^1^, Jiancheng Guo^2^, Jie Zhao^4^* ,Yonggao Zhang^3^*

^1^ Department of Ultrasound, The First Affiliated Hospital of Zhengzhou University, Zhengzhou, 450052, China

^2^ BGI College & Henan Institute of Medical and Pharmaceutical Sciences, Zhengzhou University, Zhengzhou, 450052, China

^3^ Department of Radiology, The First Affiliated Hospital of Zhengzhou University, Zhengzhou, 450052, China

^4^ Internet Medical and System Applications of National Engineering Laboratory, Zhengzhou, 450052, China

E-mail:

Yiling Yang*: fccyangyl@zzu.edu.cn

Shiqi Ren: shiqiren9766@163.com

Wenpeng Huang: hwpeng19950930@163.com

Jiahan Dong: 775364070@qq.com

Jiancheng Guo: gjc@zzu.edu. cn

Jie Zhao*: zhaojiezzu@163.com

Yonggao Zhang*: zyg01578@126.com

**Experimental Section**

1. Chemicals

Camptothecin (95 %) was obtained from Aladdin (China). MitoTracker green, 3,3’ -dioctadecyloxacarbocyanine perchlorate (DiO), dihydroethidium (DHE), TdT-mediated dUTP nick-end labeling (TUNEL), Ki 67 antibody were acquired from Sigma-Aldrich (USA). Cell counting kit 8 (CCK 8), cell cycle analysis kit, anti-CD 63 and anti-CD 9 were purchased from Beyotime (China). All reagents were used without further purification.

1. Cell culture

Hela cells and HUCEC cells were obtained from Cell Bank of the Chinese Academy of Sciences and was incubated in Dulbecco Modified Eagle Medium (DMEM; Gibco) at 37 ℃ in 5 % CO_2_.

1. Physical Characterization of NPs

The morphology of NPs was observed by transmission electron microscopy (TEM; Tecnai G2 F20 S-Twin, FEI, USA) at 100 keV acceleration voltage. The zeta potential and hydrodynamic diameter of the NPs in 1 × PBS in suspension were measured by dynamic light scattering (DLS, Nano-Zen 3600, Malvern Instruments, UK).

1. Cell viability

CCK-8 assay was used to evaluate the dark toxicity of NPs in three cell types: HUCEC, Hela and patient-derived cells. Firstly, HUCEC cells (5 Wells for each group) were cultured on 96-well plates at a density of 5×10^3^ cells per well in DMEM medium containing 10 % fetal bovine serum in a humid atmosphere containing 5 % CO_2_ at 37 ℃ for 24 h. After 24 h, the DMEM was replaced with fresh DMEM containing EC and ECC, respectively, and then incubated for 4 h. After being added 10 μL CCK 8 and incubated for 2 h, the absorbance value at the characteristic peak of 450 nm was measured by microplate reader (Rayto-6000 system, Rayto, China). Hela cells and patient-derived cells were used to further verify the effect of NPs on cell viability. 5 × 10^3^ Hela cells were seeded in in 96-well plates and cultured for 24 h, 5 wells per group, which were treated with varying groups: (1) Control; (2) RT (6 Gy); (3) CPT (50 μg); (4) EC (with an equivalent CPT dose of 50 μg); (5) ECC (with an equivalent CPT dose of 50 μg); (6) EC+RT; (7) ECC+RT. 24 h later, 10 μL CCK 8 was added and incubated for 2 h. The absorbance at 450 nm was determined by using a microplate reader (RayTO-6000 system, China, Rayto). The treatment and grouping of patient-derived cells were the same as that of Hela cells.

1. Flow cytometry of cell cycle

Patient-derived cells were incubated in a 6-well plate at 37 ℃ for 24 h and designed into three groups with two complex wells in each group: (1) control; (2) CPT (50 μg/mL); (3) EC (with an equivalent CPT dose of 50 μg/mL). After treatment and incubation for 6 h, cells in each group were collected, and the number of cells was counted to 1 × 10^6^ with 2 mL PBS. After centrifugation, 500 μL cold ethanol was added and stored overnight at 4 ℃ to achieve the effect of cell fixation. The overnight cells were then centrifuged, washed with 2 mL PBS, and 500 μL of the matching staining solution (PI: RNA enzyme = 9:1) was added to the cells. The cells were incubated at room temperature, dark for 45 min, and shaken every 5 minutes to make full contact. Flow cytometry was used to detect the cells.

1. Western blot

Western-blot analysis was performed to analyze the level of protein from cells in patient-derived exosomes and EC. The total cellular protein was extracted using a protein extraction kit (Dingguo, China). The cells were lysed in RIPA buffer containing protease inhibitors and phosphatase inhibitor (Sigma-Aldrich). Bradford protein assay (Bio-Rad Ltd., Germany) was performed to measure the concentration of protein CD 63 and CD 9. The total protein was isolated and subjected to 10 % SDS-PAGE (Bio-Rad Ltd., Germany), and transferred to a PVDF membrane (Millipore Ltd., USA). Afterward, TBST (20mMTris,150mMNaCl) with 5 % milk was used to block non-specific binding sites. The membranes were then incubated with the primary antibodies (Bethyl Laboratories, Inc.) in 4 ℃ overnight. After washing and further incubation with appropriate secondary antibodies (Jackson Immuno Research Laboratories) for 1 h at room temperature. The signal was detected using an enhanced chemiluminescence system (ECL; Amersham).

1. Colony formation assay

Hela cells were seeded into 6-well plates with 500 per well and incubated at 37 °C for 24 h. The radiation dose curve experiments were designed into three groups with two complex wells in each group: (1) control; (2) CPT (50 μg/mL); (3) EC (with an equivalent CPT dose of 50 μg/mL). After the incubation time reached 2 h, three washings were performed. One group was not irradiated by RT, and the other groups were received RT (0, 2, 4, 6, 8 Gy). After washing and changing DMEM medium, 10 days later, cells were stained with Crystal violet dye. This process calls the standard linearquadratic model, counts the colonies covering at least 50 cells, and completes the effective calculation of the colony formation rate. Then, the survival rate of the colony was calculated to realize the evaluation of the effects of various treatments. The process is repeated three times.

1. Immunofluorescence staining sections

When tumor size of mice reached approximately 200 mm^3^, the mice were divided randomly into 7 groups (each group included 5 mice): (1) Control; (2) RT (6 Gy); (3) CPT (50 μg); (4) EC (with an equivalent CPT dose of 50 μg); (5) ECC (with an equivalent CPT dose of 50 μg); (6) EC+RT; (7) ECC+RT. Radiotherapy was performed 12 h after intravenous injection. Then the fluorescent dye, DCFH-DA (10 μmol/L, 50 μL) was injected intratumorally. Subsequently, tumors from each group were dissected. The main organs and tumors were treated with TUNEL and Ki-67 staining, and observed with light microscopy for histological analysis (Olympus IX71).

1. HE staining

At day 19 of the treatment, the mice were sacrificed. The tumor and the main organs including hearts, livers, spleens, lungs and kidneys were harvest, fixed in 4 % formaldehyde and embedded in paraffin. The paraffin was sliced at stained with hematoxylin and eosin (H&E).

1. In Vivo Pharmacokinetics.

PDX tumor model was established as described above. Then the PDX tumor bearing mice were divided into two groups to intravenously inject CPT, EC and ECC respectively. The blood samples were obtained at 0.5, 2, 4, 8, 12 and 24 h after administration. After adding PBS, the samples were freeze-thawed repeatedly and ultrasonicated for 5 min. Then the samples were centrifuged at 3000 rpm and the supernatant was collected for fluorescence analysis.


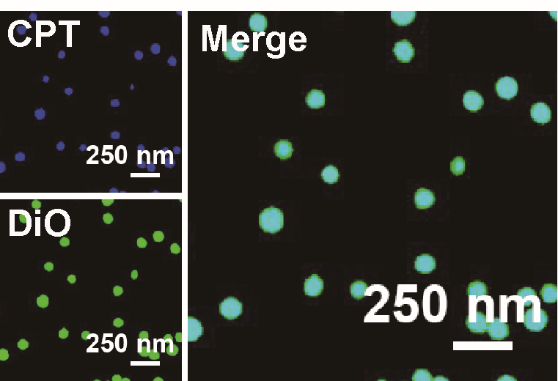


**Figure S1**. Confocal microscopic analysis of the colocalization of CPT (blue) and DiO (green) within the ECC.


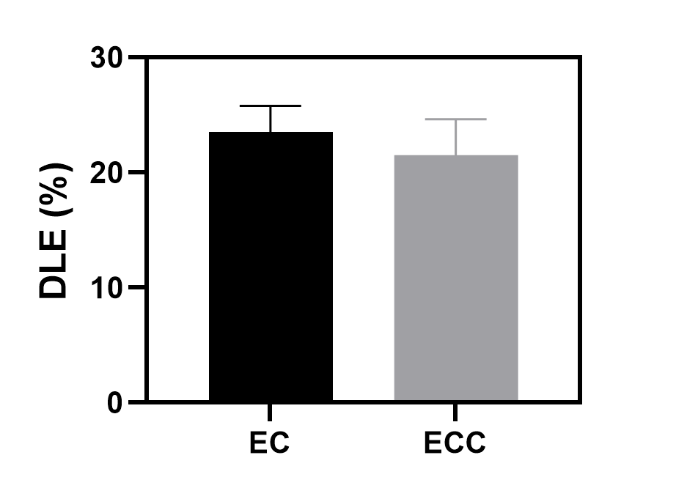


**Figure S2.** Drug loading efficiency of CPT under different loading method.


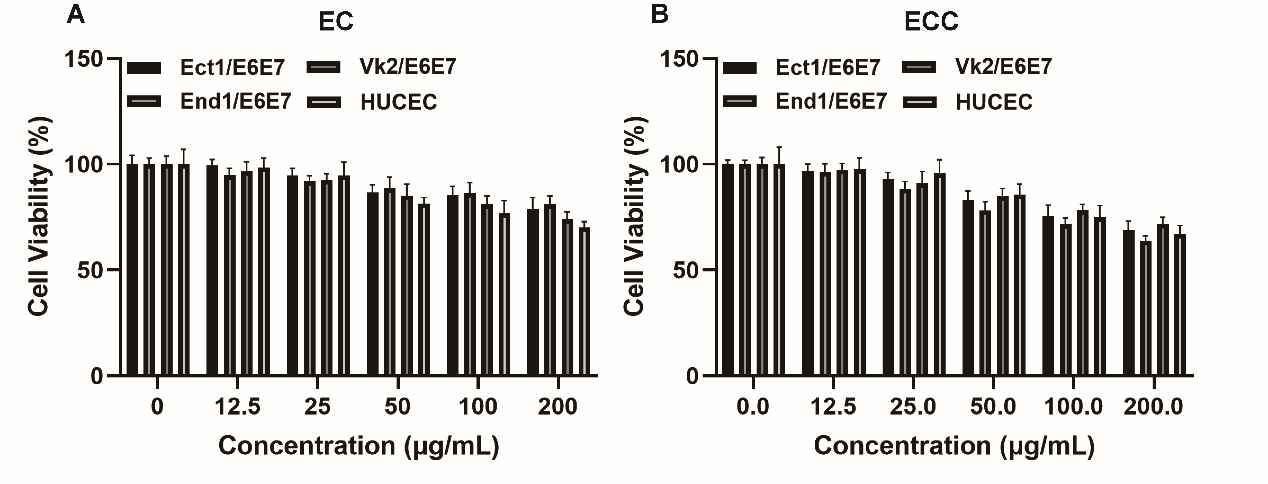


**Figure S3**. Cell viability of 4 cell lines after incubation with (A) EC or (B) ECC at various concentrations.


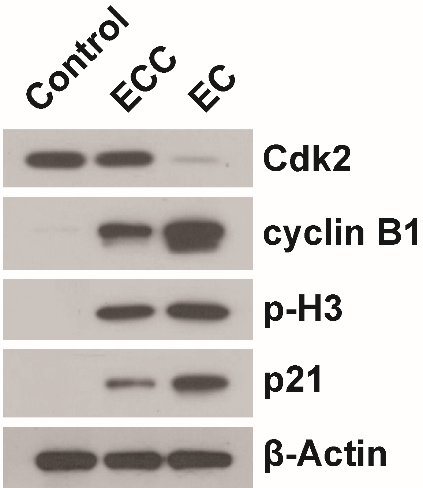


**Figure S4.** Western blot analysis of patient-derived cells after treatment with PBS, ECC and EC respectively.
